# Teaching and learning high school mathematics in the post-COVID-19 era: investigating the emotion factor

**DOI:** 10.3389/fpsyg.2025.1617765

**Published:** 2025-11-05

**Authors:** Georgios Polydoros, Alexandros-Stamatios Antoniou, Ilias Vasileiou, Charis Polydoros

**Affiliations:** ^1^Department of Mathematics and Applied Mathematics, University of Crete, Heraklion, Greece; ^2^Department of Primary Education, National and Kapodistrian University of Athens, Athens, Greece

**Keywords:** emotional engagement, online mathematics learning, high school education, post-COVID-19 era, AOEQ model

## Abstract

**Introduction:**

Emotions play a pivotal role in learning, particularly in the post-COVID-19 era where online instruction has become prevalent. Despite this, emotional engagement in online mathematics remains underexplored.

**Methods:**

The study introduces the *Assessment Online Emotions Questionnaire (AOEQ)*, developed to assess emotional dynamics among 406 10th-grade students in online mathematics. Guided by *Russell’s Circumplex Model of Affect*, data were analyzed using EFA and CFA to validate the instrument

**Results:**

The analyses confirmed a robust three-factor structure (Useful, Unuseful, Neutral Emotions) with high reliability and significant correlations between emotional states and academic performance.

**Discussion:**

Findings highlight the predictive power of positive emotions for academic success and propose AOEQ as an innovative tool for evaluating emotional engagement in virtual learning.

## Introduction

The post-COVID era has ushered in significant changes in teaching and learning practices, particularly in high school mathematics education. With the widespread adoption of remote teaching, traditional classroom dynamics have been reshaped, prompting educators and students to adapt to new digital environments (Stewart and Lowenthal, 2022; Tzafilkou et al., 2021). This transformation has necessitated a critical examination of the multifaceted factors—especially emotional dynamics—that influence student engagement and learning outcomes.

Emotional processes are fundamental to effective learning as they are closely linked to the neural circuits of the central nervous system. Foundational theories conceptualize emotions as complex reactions involving both cognitive and physiological components (Frijda and Mesquita, 1994; Scherer, 2005), while Goleman underscores the importance of emotional intelligence, comprising self-awareness, impulse control, and empathy, for academic success (Perloff, 1997). Additionally, Russell’s (1980) Circumplex Model of Affect, which organizes emotions along the dimensions of arousal and valence, offers a robust framework for understanding the spectrum of emotional experiences in educational contexts.

The shift to mandatory remote teaching in the post-COVID educational landscape has sparked notable changes in the emotional experiences of students, particularly those in high school. Recent studies indicate that the enforced transition to online learning environments has significantly altered how emotions are experienced and expressed in this age group (Bakkialakshmi and Sudalaimuthu, 2022; Cockerham et al., 2021; Lee et al., 2025). This evolving emotional landscape calls for innovative tools and methodologies to capture these dynamics and better support students’ affective needs in virtual classrooms.

In light of these developments, the primary aim of this study is to investigate the range of emotions experienced by 10th-grade students during online mathematics instruction and to examine the relationship between these emotional states and academic performance. Previous studies have primarily focused on either cognitive or technical aspects of online learning, neglecting the emotional dimensions that influence student engagement. For instance, while Bolliger and Martin (2021) emphasized cognitive engagement, they did not account for the psychological readiness of students. Similarly, studies such as Wijekumar (2021) have focused predominantly on these cognitive and technical aspects, overlooking the crucial role that emotional dimensions play in student engagement and motivation—both of which are essential for successful learning experiences. Thus, a gap remains in the literature, particularly regarding how emotional factors interact with cognitive and technical elements to influence overall learning outcomes (Wu and Yu, 2022).

Recognizing and integrating these emotional dimensions into online learning at the secondary education level can significantly enhance student engagement and foster a more holistic approach to learning. This approach can ultimately lead to improved academic performance and greater student satisfaction (Li et al., 2023). Given this need, this study aims to bridge the gap by integrating emotional engagement with academic performance in virtual mathematics education.

Guided by Russell’s model, the study is driven by the following research questions:

(1) What are the predominant emotions that students experience in online mathematics environments?
(2) How do these emotions correlate with their academic outcomes?
(3) Which interventions and strategies can enhance emotional engagement and improve learning performance?

By addressing these questions, the study seeks to bridge existing gaps in the literature and provide valuable insights for educators and policymakers in the development of more effective and emotionally supportive online learning environments.

## Literature review

### Emotional engagement in learning

Emotions play a vital role in the learning process, shaping not only cognitive functioning but also motivation and academic achievement. Research over the past decade has increasingly emphasized the importance of understanding emotional dynamics in educational settings. Positive emotions—such as joy, contentment, and curiosity—serve to enhance engagement, deepen information processing, and improve long-term memory retention (Pekrun and Perry, 2014; Fredrickson, 2016). In contrast, negative emotions—such as anxiety, frustration, and boredom—often impair cognitive performance and can lead to disengagement from the learning process (Rienties and Tempelaar, 2013; Stenbom et al., 2016).

The criticality of emotional engagement is particularly evident in online learning environments, where the lack of in-person interaction can intensify feelings of isolation and stress. As a result, emotional engagement takes on a heightened significance, as it directly impacts students’ participation, persistence, and overall performance (Bolliger and Martin, 2021). Numerous studies have demonstrated that students who report higher levels of emotional engagement are more likely to stay motivated, complete assignments on time, and achieve better outcomes in both synchronous and asynchronous online learning settings (Dixson, 2015; Martin et al., 2022).

Furthermore, emotional engagement is not a monolithic construct. It encompasses a variety of affective responses, ranging from enjoyment and enthusiasm to frustration and confusion. Bolliger and Martin (2021) highlight that emotional engagement is intertwined with cognitive and behavioral engagement, creating a complex, multifaceted construct that influences students’ learning trajectories. Similarly, Pekrun et al. (2017) noted that emotions such as pride, enjoyment, and relief often drive positive academic behaviors, while anxiety and hopelessness can undermine both effort and achievement.

Recent research has also expanded on these foundational findings by exploring the role of digital tools and strategies in fostering emotional engagement. For instance, David and Weinstein (2023), as well as previously Schöbel et al. (2022), emphasized that gamified and interactive online learning environments can significantly enhance students’ emotional engagement, motivation, and problem-solving skills. These studies demonstrated that when learning activities incorporate feedback mechanisms, goal-oriented challenges, and elements of enjoyment, students tend to experience higher levels of positive emotions and sustained engagement. Meanwhile, Stewart and Lowenthal (2022) found that well-structured instructor presence—through timely feedback, supportive communication, and active participation—significantly mitigates feelings of alienation and helps sustain emotional engagement.

### The impact of the COVID-19 pandemic on emotional engagement

The abrupt transition to remote learning during the COVID-19 pandemic highlighted the critical importance of emotional engagement in digital educational environments. With traditional in-person classes replaced by virtual classrooms almost overnight, many students found themselves grappling with new challenges. Research has consistently shown that the pandemic-induced shift to remote learning significantly heightened levels of anxiety, frustration, and isolation among learners (Leigh et al., 2021). Hendriksen et al. (2021) noted that the uncertainty surrounding the pandemic, coupled with the lack of direct social interaction, led to emotional distress that negatively influenced academic motivation and performance.

At the same time, some students experienced a degree of increased autonomy and satisfaction as they adapted to self-paced learning (Anthonysamy and Singh, 2023). Those who were able to manage their time effectively often reported a sense of control and accomplishment, which in turn enhanced their positive emotional engagement. This suggests that, for certain learners, the shift towards more independent learning fostered a greater sense of agency and motivation. However, this was not a universal experience. The benefits of autonomy appeared to depend significantly on individual circumstances, such as the level of support available at home, as well as students’ digital literacy skills. Without these conditions, some learners may have struggled to cope with the demands of self-regulation, ultimately limiting their ability to fully engage with the learning process.

The work of Acosta-Gonzaga and Ruiz-Ledesma (2022) underlined the need for targeted strategies to address the emotional dimension of online engagement during the pandemic. They found that students’ emotional states were directly linked to their ability to participate and succeed in remote instruction, making emotional engagement a critical determinant of educational effectiveness. Meanwhile, Stewart and Lowenthal (2022) emphasized the dual nature of emergency remote education (ERE): while it provided a lifeline for continuing education during a global crisis, it also reshaped student-teacher interactions in ways that presented both opportunities and challenges. These authors highlighted that the rapid deployment of ERE tools often left instructors and students unprepared to deal with the emotional consequences of online learning, underscoring the need for proactive strategies to foster emotional well-being.

The COVID-19 pandemic brought significant disruption to everyday life, with education being one of the most affected sectors. The sudden shift to online learning environments led many students to experience heightened levels of stress and anxiety (Bakkialakshmi and Sudalaimuthu, 2022). Key contributors to this emotional strain included the absence of direct interaction with teachers and classmates, limited opportunities for social connection, and the pressure of learning to navigate unfamiliar digital platforms, such as video conferencing tools. The lack of physical presence and the newness of virtual classrooms intensified feelings of loneliness and emotional disconnection among students, as traditional peer interactions and social routines were abruptly interrupted (Bakkialakshmi and Sudalaimuthu, 2022).

Nonetheless, the emotional experience of students during this time was not uniform. While some found relief from isolation through online communication tools and collaborative platforms, these alone were not enough to foster meaningful emotional engagement. Studies emphasized that a strong teacher presence, regular and personalized feedback, and structured emotional support were crucial in promoting student well-being in digital settings (Acosta-Gonzaga and Ruiz-Ledesma, 2022). Furthermore, some students adapted positively to self-directed learning during confinement. Those who developed effective time-management skills and embraced autonomous learning strategies reported greater satisfaction and a sense of accomplishment, which contributed to more positive emotional involvement in their studies. However, such benefits largely depended on each student’s digital competence and personal learning environment (Anthonysamy and Singh, 2023).

### Measuring emotional responses in online learning

Understanding emotional engagement in online learning environments requires reliable and validated instruments that capture the complex nature of students’ affective experiences. One foundational framework is Russell’s Circumplex Model of Affect, which organizes emotional experiences along two primary dimensions: valence (positive/negative) and arousal (high/low) (Russell, 1980). This model provides a structured approach for identifying and analyzing the wide range of emotions students may encounter while learning online. By mapping emotions on this circular model, researchers can examine how feelings such as excitement, contentment, anxiety, or boredom influence students’ engagement and outcomes.

Recent studies have built on this foundational framework to address the unique challenges of online education. For example, Martin et al. (2022) and Tzafilkou et al. (2021) both highlighted the importance of capturing both positive and negative emotions in online settings. Their research demonstrated that students’ emotional states directly impact their participation, motivation, and ultimately, their academic performance. By measuring a spectrum of emotions—ranging from enthusiasm and curiosity to frustration and stress—researchers have gained a more nuanced understanding of how emotional engagement shapes learning experiences.

In addition to theoretical models, practical tools have been developed to measure these emotional responses. The Assessment Online Emotions Questionnaire (AOEQ), used in this study, exemplifies such an instrument. Specifically designed for online learning contexts, the AOEQ captures useful, unuseful, and neutral emotions and links these emotional states to academic performance. This questionnaire complements other widely used scales, such as the Achievement Emotions Questionnaire (Pekrun et al., 2011), by focusing on the unique emotional dynamics of remote education. Other studies, e.g., by Bieleke et al. (2021) have utilized similar instruments to explore how emotional responses correlate with learning behaviors, student satisfaction, and final grades. These tools have proven invaluable for identifying emotional patterns that predict success or struggle in digital learning environments.

Furthermore, researchers have refined these instruments to address the nuances of emergency remote education (ERE). Stewart and Lowenthal (2022) emphasized that the rapid shift to online instruction during the pandemic required educators to better understand students’ emotional experiences in order to provide effective support. Their findings reinforced the need for comprehensive measurement tools that consider the multifaceted nature of emotions in online learning, including the interplay between cognitive engagement, emotional states, and social interaction.

### Practical implications of emotional engagement

The research consistently underscores the importance of addressing the emotional dimensions of student engagement to improve outcomes in online learning environments. As educators increasingly rely on digital platforms for instruction, understanding and supporting students’ emotional needs has become critical. One of the most effective strategies is to strengthen teacher presence in virtual classrooms (Park and Kim, 2020). demonstrated that when instructors actively interact with students, provide timely feedback, and maintain open channels of communication, positive emotional engagement increases while feelings of anxiety and frustration diminish. Park and Kim (2020) further confirmed that consistent teacher involvement contributes to a sense of trust and security, allowing students to engage more fully with the material and their peers.

Peer collaboration is another powerful intervention. By fostering opportunities for students to work together on projects, engage in group discussions, and share insights through online forums, educators can help alleviate the isolation that often accompanies remote learning. Studies by Stewart and Lowenthal (2022) have shown that structured group activities not only boost emotional engagement but also enhance critical thinking and problem-solving skills.

Clear communication about course goals, assignments, and expectations is also essential. Research by Tzafilkou et al. (2021) indicates that when students have a clear understanding of what is required of them, they experience lower levels of stress and greater confidence in their ability to succeed. Providing comprehensive syllabi, detailed grading rubrics, and regular reminders of upcoming deadlines helps students manage their workload more effectively, leading to a more positive emotional experience.

Digital literacy training and improved access to technology are additional factors that play a key role in fostering emotional engagement. Students who feel confident using online tools are less likely to encounter technical difficulties that can cause frustration or disengagement (Yu et al., 2020). Training sessions that cover basic and advanced digital skills, coupled with access to reliable devices and high-speed internet, enable students to navigate online platforms smoothly. These interventions not only reduce stress but also build a sense of competence and autonomy, further enhancing emotional engagement (Kumpikaitė-Valiūnienė et al., 2021).

Taken together, these practical strategies create an environment where students feel supported, confident, and connected. When educators understand the emotional dynamics of their students and implement interventions that enhance teacher presence, peer collaboration, clear communication, and digital literacy, they pave the way for more effective and emotionally positive online learning experiences. By doing so, they not only improve academic achievement but also contribute to the overall well-being of their students.

## Method

### Theoretical framework

This study draws on three key theoretical models to explain the role of emotions in online mathematics learning: Russell’s Circumplex Model of Affect (1980), Pekrun’s (2006) Control-Value Theory of Achievement Emotions, and Goleman’s (1995) theory of emotional intelligence.

Russell’s model organizes emotions along two dimensions—valence (pleasant–unpleasant) and arousal (high–low)—and provides the basis for categorizing students’ emotional experiences into Useful, Unuseful, and Neutral Emotions. This categorization was applied in the development of the Assessment Online Emotions Questionnaire (AOEQ), capturing a wide range of emotional responses relevant to online learning.

Neutral emotions refer to affective states of low arousal and valence (e.g., calm, bored, relaxed), while “disengaged” was used more functionally to describe lack of emotional activation, not as a separate emotional category. The AOEQ model builds upon Russell’s Circumplex by clustering emotions into three functional categories (Useful, Unuseful, Neutral) based on their observed academic impact, without disregarding the valence-arousal dimensions. This categorization was both theory-driven and empirically supported through factor analysis and regression testing.

Complementing this, Pekrun’s Control-Value Theory explains how students’ perceptions of control over tasks and the value they place on them shape their emotional experiences. According to the theory, positive emotions arise when learners feel in control and value the activity, which leads to higher engagement and performance. In contrast, negative emotions, linked to low control or value, are associated with lower motivation and academic outcomes, findings confirmed by this study.

Finally, Goleman’s emotional intelligence framework provides insight into students’ ability to recognize, regulate, and use emotions to support learning. In online settings, where self-regulation is key, students with greater emotional awareness may be better equipped to stay focused and resilient.

Together, these models explain how emotional states are generated, categorized, and related to academic performance. They form the foundation of this study’s approach to examining the emotional landscape of students in online mathematics education.

### Participants

The study involved 406 10th-grade students from online mathematics instructions. These students were selected through purposive sampling to ensure that all participants had been exposed to online mathematics instruction during the COVID-19 pandemic. Prior to the main data collection, a pilot study was conducted with a separate group of 21 students to refine the survey items and ensure clarity and reliability. None of the pilot participants were included in the final dataset. The Demographic Characteristics of the Participants are depicted in Table 1.

**Table 1:** Demographic characteristics of participants.

Characteristics	Category	Frequency	Percentage
Gender	Male	200	49.3%
	Female	206	50.7%
Age	15 years	406	100%
Digital literacy	Low (1–2)	50	12.3%
	Medium (3)	150	36.9%
	High (4–5)	206	50.8%
Technology access	High	250	61.6%
	Moderate	120	29.6%
	Low	36	8.8%
Socio-economic status	High	80	19.7%
	Medium	220	54.2%
	Low	106	26.1%
Area of residence	Urban	180	44.3%
	Suburban	150	36.9%
	Rural	76	18.7%

### Survey instrument

The primary instrument used was the Assessment Online Emotions Questionnaire (AOEQ). This tool was designed to measure three emotional dimensions: Useful Emotions, Unuseful Emotions, and Neutral Emotions (see Appendix).

- Useful emotions: included items such as “I feel contented during my online mathematics classes” and “I feel happy while studying mathematics online.”
- Unuseful emotions: captured negative emotional states, with items such as “I feel stressed about the amount of work in my course” and “I feel sad when I struggle to solve problems.”
- Neutral emotions: assessed less polarized emotional states, including items like “I feel calm when working on my assignments” and “I feel at ease when navigating the course platform.”

Each item was rated on a 5-point Likert scale, ranging from 0 (neutral) to 4 (strongly agree). In addition, students provided information on their final course grades and responded to demographic questions, such as gender, years of online learning experience, and access to technology.

### Procedure

Educators distributed an online survey link to their students, who completed the questionnaire anonymously within a designated timeframe. All responses were securely recorded without identifiable information, maintaining confidentiality.

To ensure content validity, three experts in educational psychology and e-learning reviewed the initial list of 20 items. Based on their feedback, items were revised for clarity, age appropriateness, and conceptual alignment. Four items were removed due to redundancy or overlap, resulting in the final set of 16 items used in the main study.

#### Pilot study

A pilot study involving 21 students validated the Assessment Online Emotions Questionnaire (AOEQ). Participants provided feedback on clarity, usability, and length. Minor adjustments were made to improve wording and question sequence. Reliability was confirmed with Cronbach’s alpha values exceeding 0.75, making the revised instrument suitable for the main study.

### Data coding

- Survey responses: Likert-scale answers were numerically coded (0–4) and entered into SPSS 27.
- Final grades: obtained from instructors on a 0–20 standardized scale.
- Data validation: checked for missing values and outliers before analysis.
- Grade verification: final grades were cross-checked by math teachers to ensure accuracy.

### Validity and reliability

- Validity: Exploratory Factor Analysis (EFA) using Principal Axis Factoring with promax rotation identified three factors: Useful, Unuseful, and Neutral Emotions, explaining 81.5% of variance and confirming construct validity.
- Reliability: high internal consistency was observed:
  ○ Useful emotions: *α* = 0.89
  ○ Unuseful emotions: α = 0.93
  ○ Neutral emotions: α = 0.84

### Data analysis

#### Preliminary tests for factor analysis

- Kaiser-Meyer-Olkin (KMO): confirmed adequacy of inter-item correlations.
- Bartlett’s test: verified meaningful relationships among variables.
- Anti-image and communalities: ensured individual items significantly contributed to their factors.

#### Exploratory factor analysis (EFA)

- Principal axis factoring: suitable due to non-assumption of normality.
- Eigenvalues and variance: factors with eigenvalues above 1.0 retained.
- Factor loadings: confirmed strong associations of items with respective factors.

#### Statistical tests for final grade analysis

- Assumption checks

1. Shapiro–Wilk: verified normal distribution of grades.
2. Levene’s Test: confirmed equal variances among groups.

- Demographic analysis

Included Gender, Digital Literacy, Technology Access, Socio-Economic Status, and Area of Residence to identify distribution patterns.

- Emotional dimension scores

Calculated mean scores from five items per emotional dimension, resulting in three continuous variables per student: Useful, Unuseful, Neutral Emotions.

- Implemented statistical tests

1) One-Way ANOVA: assessed academic performance differences among emotional dimensions.
2) Correlation analysis: measured relationships between emotional mean scores and final grades.
3) Multiple regression analysis: evaluated unique contributions of emotional dimensions and demographics on academic performance.

#### Justification of statistical methods

- Shapiro–Wilk and Levene’s tests validated assumptions for parametric analysis.
- Descriptive statistics contextualized demographic variables.
- Pearson Correlation identified linear relationships.
- Multiple Regression controlled for interdependencies among variables.
- One-Way ANOVA identified significant emotional impact.
- Tukey’s HSD post-hoc clarified specific differences between emotional categories.

## Results

Before proceeding with the factor analysis, several preliminary statistical tests were conducted to confirm the suitability of the data. The Kaiser-Meyer-Olkin (KMO) measure of sampling adequacy was 0.92, which is well above the commonly recommended threshold of 0.6. Bartlett’s Test of Sphericity was significant (χ^2^ (105) = 600.95, *p* < 0.05), indicating that the correlation matrix was not an identity matrix and that sufficient inter-item correlations existed for factor analysis. In addition, all the diagonals of the anti-image correlation matrix were above 0.5, and the communalities for all items were above 0.7 (see Appendix). Based on these overall indicators, factor analysis was deemed suitable for the 16 items (Fabrigar et al., 1999).

An Exploratory Factor Analysis (EFA) using Principal Axis Factoring with a promax rotation yielded a three-factor solution corresponding to the emotional dimensions of the study. The extracted factors explained 40.022% of the variance for Unuseful Emotions, 27.553% for Useful Emotions, and 13.923% for Neutral Emotions, as shown in Table 2.

**Table 2:** Eigenvalues and total variance explained for the final three-factor structure.

Factor	Initial eigenvalues			Extraction sums of squared loadings			Rotation sums of squared loadings		
	Total	% of variance	Cumulative %	Total	% of variance	Cumulative %	Total	% of variance	Cumulative %
1. Unuseful	6.404	40.022	40.022	6.254	39.086	39.086	4.731	29.570	29.570
2. Useful	4.408	27.553	67.575	4.142	25.887	64.973	4.229	26.429	55.999
3. Neutral	2.228	13.923	81.498	2.006	12.540	77.513	3.442	21.514	77.513

Moreover, the three factors were acceptably correlated, as shown in Table 3. The correlation between Unuseful and Useful was 0.543; between Useful and Neutral was 0.385; and between Unuseful and Neutral was 0.448.

**Table 3:** Factor correlation matrix.

Factor	1	2	3
1. Unuseful	—		
2. Useful	0.54**	—	
3. Neutral	0.45**	0.39**	*—*

** *p* < 0.001.

**Figure 1:**
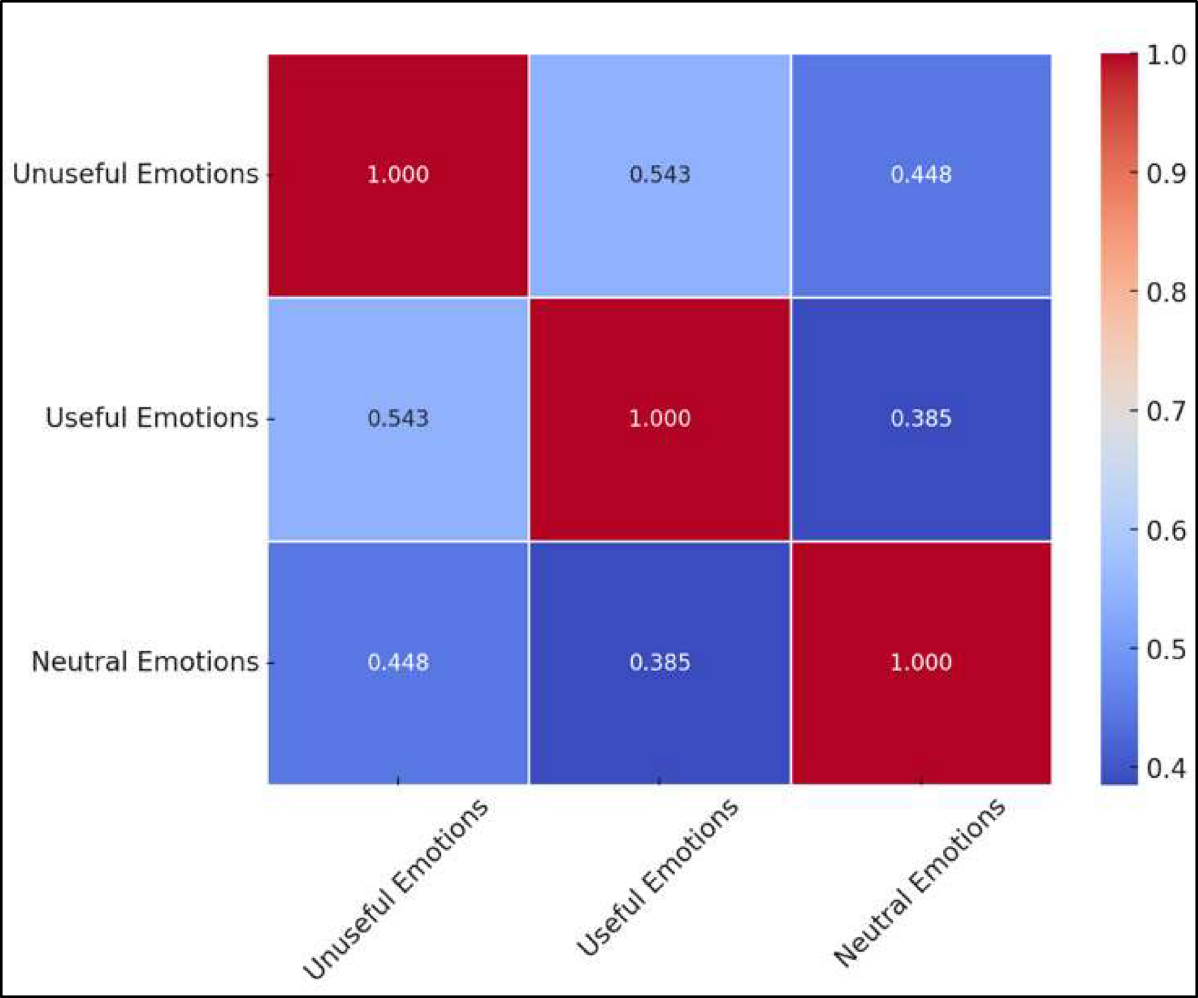
Correlation matrix of emotional dimensions in online mathematics learning.

The heatmap (Figure 1) visualizing the correlation matrix of emotional factors provides a clear representation of the relationships between the three key emotional dimensions examined in the study. It reveals that Unuseful Emotions and Useful Emotions have a moderate positive correlation (r = 0.543), suggesting that students who experience more negative emotions tend to report fewer positive emotions. Additionally, Useful Emotions and Neutral Emotions exhibit a weaker correlation (r = 0.385), indicating that students with neutral emotional states are somewhat less likely to experience strong positive emotions. Meanwhile, Unuseful Emotions and Neutral Emotions have a correlation of r = 0.448, meaning that even students who report neutral emotional responses may still experience some levels of negative emotions.

This heatmap (Figure 1) provides an intuitive visualization of how different emotional dimensions interact in online mathematics learning. The moderate correlations suggest that while these emotional factors are interconnected, they are not entirely overlapping, confirming that emotions in learning environments are complex and multidimensional. Moreover, this visualization complements the Exploratory Factor Analysis (EFA) variance explained chart, reinforcing the importance of studying these emotional dimensions separately rather than treating them as a single factor.

Reliability analysis was also performed. The first 5-item factor, labeled Useful Emotions, had a Cronbach’s alpha reliability of 0.89; the second 6-item factor, labeled Unuseful Emotions, had a Cronbach’s alpha reliability of 0.93; and the third 5-item factor, labeled Neutral Emotions, had a Cronbach’s alpha reliability of 0.84. These values indicate acceptable internal consistency (Nunnally and Bernstein, 1994), as shown in Table 4.

**Table 4:** Cronbach’s alpha for each factor of the AOEQ.

Factor	Cronbach’s alpha	Cronbach’s alpha (standardized)	Number of items
Useful emotions	0.890	0.890	5
Unuseful emotions	0.930	0.928	6
Neutral emotions	0.840	0.838	5

The factor loadings for each factor are presented in Table 5.

**Table 5:** Factor loadings for the final three-factor structure of the AOEQ.

Factor	Item	Useful emotions	Unuseful emotions	Neutral emotions
Useful emotions	1. Happy	0.81	0.25	0.29
	5. Contented	0.82	0.32	0.30
	14. Amused	0.80	0.35	0.33
	3. Elated	0.79	0.28	0.31
	15. Alert	0.78	0.30	0.27
Unuseful emotions	2. Upset	0.31	0.85	0.43
	8. Nervous	0.35	0.78	0.30
	11. Tense	0.30	0.90	0.34
	6. Sad	0.29	0.88	0.27
	4. Depressed	0.28	0.82	0.26
	16. Stressed	0.32	0.79	0.12
Neutral emotions	7. Calm	0.27	0.29	0.85
	9. At Ease	0.30	0.32	0.74
	10. Relaxed	0.31	0.30	0.86
	12. Serene	0.28	0.26	0.76
	13. Bored	0.25	0.33	0.88

The exploratory factor analysis revealed a robust three-factor structure that effectively captured the emotional landscape of online learning. Specifically, the analysis identified three distinct dimensions: Useful Emotions (e.g., Contented, Alert, Elated, Happy, Excited), Unuseful Emotions (e.g., Sad, Stressed, Depressed, Nervous, Tense, Upset), and Neutral Emotions (e.g., Calm, Serene, Relaxed, Bored, At Ease). These factors, which explained a substantial portion of the variance, underscore the multifaceted nature of emotional responses in online learning environments.

These preliminary tests and reliability analyses confirm that the data are well-suited for factor analysis, and that the AOEQ reliably measures the three dimensions of emotional experience—Useful, Unuseful, and Neutral—in online mathematics environments.

To verify the adequacy of the three-factor structure identified through Exploratory Factor Analysis, a Confirmatory Factor Analysis (CFA) was conducted using maximum likelihood estimation. The CFA results demonstrated excellent model fit, providing strong support for the structural validity of the Assessment Online Emotions Questionnaire (AOEQ). Specifically, the Root Mean Square Error of Approximation (RMSEA) was 0.048, which is below the widely accepted cutoff of 0.06, indicating a close fit of the model to the data (Clark and Bowles, 2018). The Comparative Fit Index (CFI) was 0.97, surpassing the recommended threshold of 0.95, and the Tucker-Lewis Index (TLI) was 0.96, further confirming the suitability of the three-factor solution. These indices collectively reinforce the robustness of the emotional structure underlying the AOEQ and its utility as a reliable tool for capturing students’ emotional responses in online mathematics learning environments.

### Distribution of emotional responses in online learning

**Figure 2:**
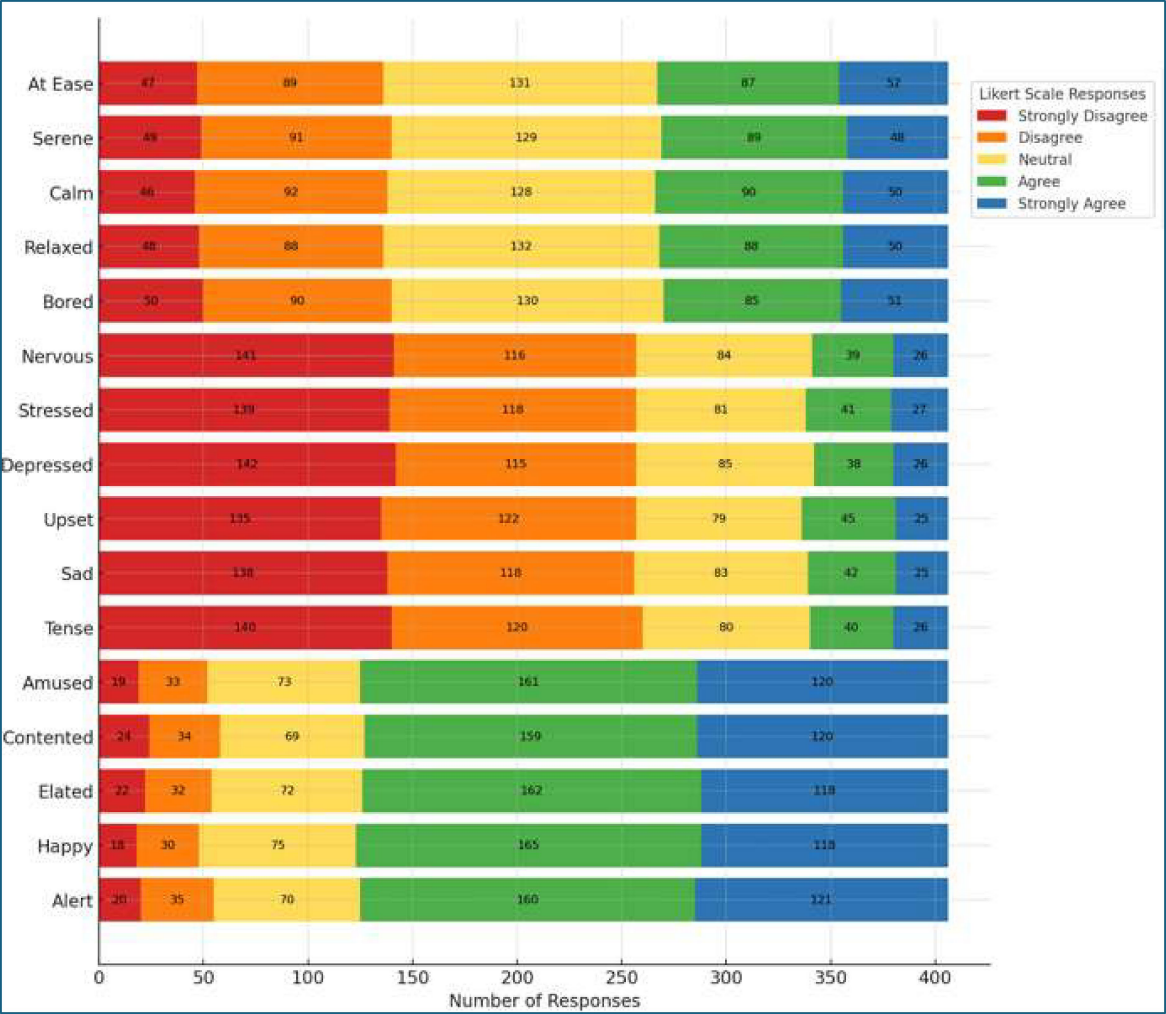
Stacked bar chart for students’ emotional responses.

The stacked bar chart illustrates the distribution of student responses across the three emotional dimensions, Useful, Unuseful, and Neutral Emotions (Figure 2).

For Useful Emotions, the majority of responses were concentrated in the “Agree” (ranging from 159 to 165 responses per item) and “Strongly Agree” (118 to 121), indicating that most students experienced positive emotional states during online mathematics learning.

In contrast, Unuseful Emotions received substantially more responses in the “Disagree” (115 to 122) and “Strongly Disagree” categories (135 to 142), suggesting that negative emotions were reported by relatively fewer students.

Neutral Emotions showed a more balanced distribution, with most responses falling within the “Neutral” range (128 to 132), reflecting a moderate or emotionally indifferent state among a substantial portion of students.

### Preliminary statistical tests

Before conducting further analyses, the following tests were performed on the final grade data (*N* = 406):

- Normality: the Shapiro–Wilk test yielded a *p*-value of 0.334 > 0.05. This indicates that the final grade data are normally distributed, with no significant outliers.
- Homogeneity of variance: Levene’s test produced a *p*-value of 0.30, confirming that the assumption of homogeneity of variances is met.

### Descriptive statistics and correlations

Table 6 presents the means, standard deviations, and Pearson correlations among the main study variables: Useful Emotions, Unuseful Emotions, Neutral Emotions, and Final Grades. As shown, students reported high levels of Useful Emotions (M = 7.49, SD = 0.88) and low levels of Unuseful Emotions (M = 2.51, SD = 0.88), while responses for Neutral Emotions were more moderate (M = 4.99, SD = 1.18). The average final grade was 9.60 (SD = 1.68), indicating overall satisfactory academic performance.

**Table 6:** Means, standard deviations, and correlations among study variables (*N* = 406).

Variable	*M*	SD	1	2	3	4
1. Useful emotions	7.49	0.88	—			
2. Unuseful emotions	2.51	0.88	−0.12*	—		
3. Neutral emotions	4.99	1.18	0.04	0.01	—	
4. Final grades	9.60	1.68	0.56**	−0.37**	0.03	—

*p < 0.05, **p < 0.001.

Useful Emotions were positively and significantly correlated with Final Grades (r = 0.56, *p* < 0.001), suggesting that students who experienced more positive emotions during online learning tended to perform better academically. In contrast, Unuseful Emotions were negatively correlated with Final Grades (r = −0.37, *p* < 0.001), indicating that negative emotional states were associated with lower academic outcomes. Neutral Emotions showed no meaningful correlation with grades (r = 0.03, *p* = 0.492), reflecting their minimal predictive value in this context.

These results highlight the strong role of emotional engagement—particularly positive affect—in supporting student success, while also confirming that negative emotional experiences can hinder academic achievement.

### One-way ANOVA results

A one-way ANOVA was conducted to assess the differences in final grades across the emotional categories (Useful, Unuseful, and Neutral). Table 7 presents the results of a one-way ANOVA assessing differences in students’ final grades across emotional categories (Useful, Unuseful, Neutral Emotions). The analysis revealed statistically significant differences among the emotional groups, F(2, 1,215) = 810.88, *p* < 0.001, with a strong effect size indicated by η^2^ = 0.57. This result means that approximately 57% of the variation in students’ academic performance can be attributed to their emotional experiences during online learning.

**Table 7:** One-way ANOVA results comparing final grades by emotional categories.

Measure	Useful		Neutral		Unuseful		F(2, 1,215)	η^2^
	*M*	SD	*M*	SD	*M*	SD		
Final Grades	10.15	1.19	8.59	1.71	7.24	1.87	810.88***	0.57

***p < 0.001, η^2^ = 0.57 indicates a large effect size (57% of variance explained by emotional category).

### Tukey’s HSD post-hoc test

Tukey’s HSD Post-Hoc test clarified significant pairwise differences in final grades among the three emotional categories (Table 8). Students experiencing Useful Emotions significantly outperformed those with Neutral (Mean Difference = 1.56, *p* < 0.001) and Unuseful Emotions (Mean Difference = 2.91, *p* < 0.001). Additionally, students reporting Neutral Emotions achieved significantly higher grades compared to those experiencing Unuseful Emotions (Mean Difference = 1.35, *p* < 0.001). These results highlight the substantial positive impact of positive emotional experiences on academic performance and emphasize the negative implications of experiencing predominantly negative emotions. The small standard errors (SE) across comparisons indicate high precision in the estimated mean differences and strengthen the reliability of the observed effects (see Table 9).

**Table 8:** Tukey’s HSD post-hoc comparisons for final grades by emotional categories.

Comparison	Mean difference	SE	*p*	95% CI
Useful vs. Unuseful	2.91	0.09	<0.001	[2.73, 3.10]
Useful vs. Neutral	1.56	0.08	<0.001	[1.38, 1.74]
Neutral vs. Unuseful	1.35	0.10	<0.001	[1.14, 1.56]

All pairwise comparisons are statistically significant at *p* < 0.001.

**Table 9:** Multiple regression coefficients predicting final grades.

Predictor	B	SE	t	*p*	95% CI
Useful emotions	0.71	0.09	8.26	0.000	[0.54, 0.88]
Unuseful emotions	−0.43	0.08	−5.34	0.000	[−0.59, −0.27]
Neutral emotions	0.14	0.08	1.75	0.081	[−0.02, 0.30]
Gender	0.19	0.05	3.92	0.000	[0.10, 0.28]
Digital literacy	0.11	0.02	6.66	0.000	[0.08, 0.14]
Technology access	0.11	0.02	6.54	0.000	[0.08, 0.14]
Socio-economic status	0.10	0.02	5.87	0.000	[0.06, 0.13]
Area of residence	−0.24	0.03	−8.11	0.000	[−0.30, −0.18]

Given that emotional experiences accounted for 57% of the variation in academic performance, a substantial 43% remains unexplained. To further investigate which additional factors, such as demographic variables, might contribute to explaining this remaining variance, a multiple regression analysis was conducted. This analysis aimed to identify the unique contribution of each predictor beyond emotional dimensions.

### Multiple regression

The multiple regression model was performed to assess the unique predictive power of emotional dimensions (Useful, Unuseful, Neutral Emotions) on students’ final grades, while controlling for key demographic variables. This was necessary to ensure that the observed effects of emotions were not simply confounded by contextual or personal background factors.

#### Emotional predictors

- Useful emotions (e.g., happiness, excitement) emerged as the strongest positive predictor (B = 0.71, *p* < 0.001), aligning with prior literature suggesting that students who feel more enthusiastic and confident during learning tend to perform better academically.
- Unuseful emotions (e.g., stress, anxiety) significantly decreased final grades (B = −0.43, *p* < 0.001), underscoring the negative cognitive impact of emotional distress.
- Neutral emotions showed a small, nonsignificant effect (*p* = 0.081), suggesting emotional detachment neither helps nor hinders learning outcomes in this context.

#### Demographic predictors

The analysis confirmed that several demographic variables had significant associations with academic performance, justifying their inclusion in the model.

- Gender had a small but significant effect (B = 0.19, *p* < 0.001), indicating that female students (coded as 1) tended to score slightly higher than their male peers.
- Digital Literacy and Technology Access were both strong positive predictors (B = 0.11 each, *p* < 0.001), revealing that students who feel confident in using technology and have better access to devices and connectivity perform better in online learning environments.
- Socio-Economic Status (SES) also showed a notable effect (B = 0.10, *p* < 0.001), reflecting how financial and educational resources at home may enhance learning conditions and support.
- Area of Residence emerged as a significant negative predictor (B = −0.24, *p* < 0.001), with students from rural areas scoring lower than those from urban or suburban regions. This result may be attributed to inequalities in infrastructure, internet access, or teacher support in remote communities.

The multiple regression model predicting students’ final grades is:


$$\begin{array}{c} \mathrm{Final Grade}=4.22+0.71\times \mathrm{Useful Emotions}–0.43\\ \times \mathrm{Unuseful Emotions}+0.14\times \mathrm{Neutral Emotions}\\ +0.19\times \mathrm{Gender}+0.11\times \mathrm{Digital Literacy}+0.11\\ \times \mathrm{Technology Access}+0.10\times \mathrm{Socio}\\ -\mathrm{Economic Status}–0.24\times \mathrm{Area of Residence}+\epsilon \end{array}$$

All predictors were entered simultaneously in the regression model. While Useful Emotions, Unuseful Emotions, and all demographic variables were statistically significant predictors (*p* < 0.05), the variable Neutral Emotions was not statistically significant (*B* = 0.14, *p* = 0.081).

This indicates that while positive and negative emotions directly influenced academic outcomes, neutral emotional states did not show a consistent or meaningful contribution to final grade prediction. The inclusion of Neutral Emotions in the model, however, ensures that the analysis fully accounts for the spectrum of emotional experiences during online learning.

The multiple regression analysis confirmed that emotional experiences are robust predictors of academic success, even when demographic influences are accounted for. Notably, Useful Emotions and demographic advantages (like digital literacy and SES) boost performance, while Unuseful Emotions and living in rural areas pose risks to academic achievement. These findings highlight the importance of designing emotionally supportive and digitally inclusive online learning environments, especially for underserved student populations.

## Discussion

Having established the robust factor structure of students’ emotional dimensions—useful, unuseful, and neutral—and their statistically significant relationship with academic outcomes, the results of this study offer detailed answers to the research questions posed at the outset. Emotional engagement clearly plays a central role in students’ performance in online mathematics education, especially during and after the transition prompted by the COVID-19 pandemic.

First, this study confirms that emotional responses significantly influence academic performance in online mathematics learning. Drawing on Russell’s (1980) Circumplex Model of Affect and using the Assessment Online Emotions Questionnaire (AOEQ), results demonstrated that students who reported predominantly useful (positive) emotions—such as being happy, elated, contented, amused, and alert—achieved significantly higher final grades compared to their peers. This finding aligns with prior research by Pekrun and Perry (2014) and Fredrickson (2016), who emphasized that positive affect enhances attention, cognitive flexibility, and persistence. It also supports the Control-Value Theory (Pekrun, 2006), which proposes that students who value their academic tasks and perceive them as controllable are more likely to experience emotions that promote engagement and achievement.

Second, this study extends the existing literature by identifying that unuseful (negative) emotions, such as stress, nervousness, sadness, and being upset, were associated with lower academic performance. These findings resonate with those of Sasidharan and Kareem (2023) and Bakkialakshmi and Sudalaimuthu (2022), who highlighted the demotivating effects of negative emotional states on students’ ability to focus and persist in learning tasks. In line with Lee et al. (2025) and Vezne et al. (2022), the study shows that the pandemic-induced shift to online learning triggered stress and confusion in some students, undermining their academic self-efficacy.

Third, while neutral emotions (e.g., calm, at ease, bored) did not significantly predict academic performance, their presence in the emotional profiles of many students points to emotional disengagement. As Wu and Yu (2022) and Rienties and Tempelaar (2013) suggest, such neutral states may not be harmful per se but indicate a lack of emotional arousal, which could result in lower motivation and suboptimal academic outcomes if unaddressed. This finding underscores the importance of recognizing emotional ambivalence as a hidden barrier to learning, especially in digital contexts.

Fourth, the study found that positive emotions were the most frequently experienced. The stacked bar chart analysis showed that the majority of students selected “Agree” or “Strongly Agree” for useful emotion items, while unuseful emotions were more often rated as “Disagree” or “Strongly Disagree.” This pattern suggests that students were generally able to engage with online math in emotionally constructive ways, especially when instructional design was supportive. This observation echoes findings by Cockerham et al. (2021), who found that self-paced learning supported autonomy and enthusiasm, and by Stewart and Lowenthal (2022), who emphasized the role of timely feedback and teacher presence in maintaining emotional engagement.

Fifth, the study contributes to the literature by introducing a three-dimensional model (useful, unuseful, and neutral) that extends Russell’s two-axis structure. This approach more accurately captures the emotional complexity of students in online mathematics environments, where engagement is shaped not only by arousal and valence but also by context-specific interactions with technology, instruction, and peer dynamics.

Sixth, the Multiple Regression Analysis confirmed that emotional dimensions, especially useful and unuseful emotions, significantly predicted academic performance, even when controlling for demographic variables (e.g., gender, digital literacy, socio-economic status, and area of residence). The strong contribution of emotional dimensions affirms that emotions are not merely peripheral experiences but core determinants of learning outcomes (Fredrickson, 2016; Martin and Bolliger, 2018). Interestingly, Neutral Emotions did not significantly predict final grades, further supporting the idea that emotional detachment neither enhances nor strongly inhibits learning, but may still represent an intervention opportunity (Bolliger and Martin, 2021).

Finally, the results align with studies by Xie et al. (2021) and Tzafilkou et al. (2021), which showed that digital literacy and technology access mitigate emotional frustration. In this study, students with better tech access and digital competence experienced fewer barriers to emotional and academic engagement. This emphasizes that infrastructure and training are not merely logistical issues, they are emotional supports that influence students’ learning confidence and persistence.

### Practical implications

This section addresses the third research question, identifying evidence-based interventions and strategies that can enhance students’ emotional engagement and improve learning outcomes in online mathematics instruction.

Thε research suggests actionable strategies to foster emotional engagement:

- Teacher presence: as noted by Stewart and Lowenthal (2022) and Park and Kim (2020), regular communication and feedback reduce alienation and promote emotional well-being.
- Peer collaboration: group discussions and co-learning opportunities encourage positive emotional exchange and reduce isolation (Cockerham et al., 2021; Anthonysamy and Singh, 2023).
- Clear structure: transparent expectations, rubrics, and pacing help prevent emotional disorientation (Tzafilkou et al., 2021).
- Digital training: equipping students with technological skills prevents unuseful emotional states stemming from frustration and confusion (Xie et al., 2021; Martin and Bolliger, 2018).
- Socio-emotional support: as highlighted by Sasidharan and Kareem (2023), training in emotional regulation and stress management directly supports positive emotional functioning and academic performance.

In sum, the results of this study reinforce the theoretical and practical understanding that emotions in online learning are not peripheral, but central to educational success. The distinctions among useful, unuseful, and neutral emotions are empirically and conceptually valid, offering educators targeted insights into how emotional states shape students’ cognitive investment, motivation, and achievement. By integrating emotional dimensions with demographic and contextual factors, this study adds to the growing recognition that emotional readiness is not an “extra” in online learning—it is essential.

## Limitations and future work

Despite its valuable insights, this study has some limitations. The sample focused exclusively on 10th-grade students and the subject of mathematics, which may limit the generalizability of the findings to other age groups or disciplines. However, existing research suggests that the core emotional dimensions of engagement are likely to extend beyond this specific context to other educational settings and populations (Bolliger and Martin, 2021; Martin et al., 2022; Pekrun et al., 2014; Rienties and Rivers, 2014; Tzafilkou et al., 2021).

Notably, the structure of the AOEQ—grounded in Russell’s valence-arousal model and empirically validated through factor analysis and regression—offers potential for adaptation across subject areas beyond mathematics. Future studies could examine how emotional engagement manifests in disciplines such as science, language arts, or the humanities, potentially uncovering domain-specific emotional patterns and validating the cross-curricular applicability of the tool.

Second, the study primarily relied on self-reported data through the Assessment Online Emotions Questionnaire (AOEQ). Although this instrument proved effective, self-reports can be influenced by participants’ subjective perceptions or recall biases. Incorporating additional data collection methods—such as direct behavioral observations, interviews, or physiological measures—could provide a more comprehensive understanding of how emotions influence academic engagement.

Finally, while the study revealed statistically significant correlations between emotional dimensions and academic outcomes, it did not establish causal relationships. Further work could adopt experimental or longitudinal designs to investigate whether targeted interventions, such as enhanced teacher presence, digital literacy training, or structured peer collaboration, can directly shape students’ emotional trajectories and academic success over time.

## Conclusion

This study underscores the pivotal role of emotional experiences in shaping academic performance in online mathematics education. By investigating the experiences of high school students in online mathematics courses, it was shown that emotional engagement is not a peripheral factor but a central driver of student success.

A notable contribution of this study is the introduction of the AOEQ (Assessment Online Emotions Questionnaire), which provides a more nuanced perspective on emotional engagement. Unlike traditional models, the AOEQ distinguishes between useful, unuseful, and neutral emotions, offering a richer framework for understanding how various emotional states influence learning outcomes. This new model expands on existing theoretical frameworks by emphasizing the diversity of emotional responses and their direct correlations with academic performance. As such, the AOEQ sets a foundation for future research aimed at measuring and improving emotional engagement in online education.

The findings revealed that Useful Emotions (e.g., happiness, alertness, contentment) strongly and positively correlate with higher academic achievement. In contrast, Unuseful Emotions (e.g., stress, sadness, nervousness) were associated with lower performance, while Neutral Emotions showed no significant impact. These results were consistent across statistical analyses, including ANOVA, correlation, and multiple regression.

Demographic variables, particularly gender, digital literacy, and area of residence, also played significant roles, reinforcing that emotional engagement must be understood within the broader context of students’ lived realities and access to educational resources.

Practically, the study offers actionable recommendations: fostering teacher presence, enhancing digital infrastructure, promoting peer collaboration, and integrating emotional support into mathematics instruction. These strategies can help maximize the benefits of positive emotional engagement while minimizing the negative effects of emotional disengagement.

In sum, this research contributes to a growing body of literature emphasizing that cognitive learning in virtual environments is deeply intertwined with emotional readiness. By recognizing, measuring, and addressing students’ emotional states, educators can create more effective and inclusive online learning experiences that promote both academic performance and student well-being.

## Data Availability

The raw data supporting the conclusions of this article will be made available by the authors, without undue reservation.

## Appendix

**Table A1:** Factor emotion descriptor and survey items.

Factor	Emotion descriptor	Survey item
Useful emotions	Contented	“I feel contented during my online mathematics classes.”
	Alert	“I feel alert and ready to learn in my online classes.”
	Elated	“I feel elated when I understand a difficult problem.”
	Happy	“I feel happy while studying mathematics online.”
	Excited	“I feel excited about the topics covered in class.”
Unuseful emotions	Sad	“I feel sad when I struggle to solve problems.”
	Stressed	“I feel stressed about the amount of work in my course.”
	Depressed	“I feel depressed when I cannot keep up with lessons.”
	Nervous	“I feel nervous before attempting assignments.”
	Tense	“I feel tense during live online sessions.”
	Upset	“I feel upset when I get a low grade on an assignment.”
Neutral emotions	Calm	“I feel calm when working on my assignments.”
	Serene	“I feel serene during quiet study periods.”
	Relaxed	“I feel relaxed while reviewing course materials.”
	Bored	“I feel bored when the lecture is too long.”
	At ease	“I feel at ease when navigating the course platform.”
